# Cues, clues and the cognitivisation of perception: Do words matter?

**DOI:** 10.1177/03010066221080617

**Published:** 2022-03-11

**Authors:** Brian Rogers

**Affiliations:** University of Oxford, UK

For over one hundred years, we have been using the words “cue” and “clue” to describe the different sources of information we use to perceive the structure and layout of the surrounding world (Titchener, 1910; see Harper & Boring, 1948; Rogers, 2017). Both words have the connotation of insufficiency, incompleteness and possibly ambiguity. Cue is typically defined as a hint or a prompt, whereas a clue is defined in the Cambridge Dictionary as “*some* information *that* helps *you to* find *the* answer *to a* problem.” The idea of a cue is often attributed to Helmholtz but in the original German edition of the “Handbook of Physiological Optics,” he uses the word “Zeichen”—a “sign” (rather than a “cue”)—but note that the word “sign” also has a connotation of insufficiency or incompleteness.^1^

While the use of words like “cue” and “clue” might seem quite harmless, they represent what has been referred to as the *cognitivisation of perception*—the need to invoke “higher-level, cognitive processes” in order to explain what we see (see Tallis, 2003; Pagel, 2019). In the case of depth perception, for example, it is often argued that we need to make “*assumptions*” in order to use the available information. Specifically, we need to “assume” the homogeneity of the size of the texture elements that cover the surface in order to use texture gradient information. As humans, we are clearly capable of making assumptions and we can discuss those assumptions using language but in what sense do humans, or indeed other animals, need to make “assumptions” in order to use texture gradient information? The perspective characteristics of the spatio-temporal patterns of light reaching our eyes—the optic arrays—are all consequences of projective geometry—the sizes of objects or features in the world vary inversely with the viewing distance: that is, Euclid's law. Hence it seems more likely that the mechanisms in our visual systems have evolved to incorporate these projective properties of our visual world, rather than requiring the perceiver to make “assumptions.”

Helmholtz is also credited (correctly) with making the distinction between “primary” and “secondary” cues to depth and distance. He believed that some of the depth cues—for example, the vergence angle of the eyes, the accommodation state of the lens, and the small differences between the images reaching the two eyes—were “primary,” whereas other cues, such as perspective, shading, and height-in-the-visual-field, were “secondary.” For Helmholtz, the use of the “secondary” cues depends on experience and, as a consequence, those cues: “*enable us merely to form some **idea as to the distance** …*” In contrast, the “primary” cues lead to “*… an actual **perception** of distance.*”^2^ (p. 282) (Southall's emphasis). Many present-day textbooks on perception still refer to this distinction. Secondary cues are often referred to as “painters” or “pictorial” cues on the grounds that they represent the techniques that artists can use to give an impression of depth and distance in their flat paintings.

Is the distinction between “primary” and “secondary” cues justified? In some textbooks, “primary” cues are referred to as “physiological” (e.g., Rock, 1984) but this descriptor is surely mistaken. All sources of information about the 3D world require a physiological mechanism to extract that information. Having said that, two of Helmholtz's primary cues—the *vergence* angle of eyes and the *accommodative state* of the lens—are different from the other cues in that the information they provide comes from proprioceptive or motor signals, rather than from the characteristics of the images reaching our eyes. In the case of the *vergence* cue, it should be possible, in principle, to monitor the extent to which our eyes converge or diverge when we are looking at a particular object, and to use this angle to estimate the distance of that object (assuming that we “know” the interocular separation of our two eyes). In other words, the eyes could be acting as a range finder. The empirical evidence suggests that humans are able to use the vergence angle of the eyes, in isolation, to estimate absolute distance but the precision of those estimates, and the range of distances over which the vergence angle is useful, are both limited. Similarly, we could, in principle, monitor the *accommodative state* of the lenses in the two eyes—the extent to which the lens in the eye is flattened or bulging—to estimate the distance of a particular object, but the evidence is also weak.

Is there any justification for making a distinction between the remaining two *primary* cues—binocular disparities and motion parallax—and the so-called *secondary* cues? Projective geometry shows that the differences between the optic arrays reaching the two eyes—the binocular disparities—provide information^3^ about the locations of objects in space (assuming that we “know”^4^ the interocular separation of the eyes). Similarly, projective geometry shows that the changes in the optic array reaching a single moving eye over time—motion parallax—provide information about the locations of objects in space (assuming that we “know”^4^ how far the eye has moved). In other words, it is *geometry* that provides a sound basis (or computational theory) for the use of both binocular disparities and motion parallax. But are *secondary* cues any different? Linear perspective, texture gradients, the height-in-the-visual field, the gradient of foreshortening and occlusion are also consequences of projective geometry. The similarity becomes obvious when we refer to the “primary” cue of binocular disparity as *binocular perspective*—that is, the different perspective views of the world from two, slightly different vantage points and when we refer to the “primary” cue of motion parallax as *motion perspective*—that is, the continuously changing perspective view of the world when the head moves.

As a consequence, I see no good reason to make a distinction between “primary” and “secondary” cues in terms of the nature of the available information, that is, the underlying computational theory, but this does not mean that there are no differences in the implementation and effectiveness of the different “cues” *in practice*. Ever since Wheatstone's invention of the stereoscope in the 1830s, binocular disparities have been regarded as the most important and effective source of information *in practice* and, more recently, the TV manufacturers tried to convince us to buy so-called “3-D TVs” on the grounds that they provide the two eyes with two slightly different, disparate images. Note that the label itself carries the implication that the images presented on conventional TVs do *not* provide 3D information! But what is the evidence that binocular disparities are more powerful or more effective compared with what are regarded as “secondary” cues? First, the synoptic viewing of flat paintings (Koenderink et al., 1994) evokes a strong impression of depth and 3D structure in spite of the fact that the binocular disparities of all features in the scene are the same. Second, when the pattern of binocular disparities and the (traditional) perspective information specify opposite and contradictory 3D structures, as in Patrick Hughes’ Reverspective artworks, perspective wins out, unless the observer is standing very close to the artwork (Papathomas, 2007; Rogers & Gyani, 2010). The power of perspective is further demonstrated by the finding that when the observer moves from side-to-side while viewing a Reverspective: the motion parallax transformation is “interpreted”^4^ in accordance with the perspective information, such that the 3D structure appears to rotate *with* the observer's head movements (Rogers & Gyani, 2010).

A second reason for rejecting the idea of depth “cues” is that we don't talk about “cues to colour” or cues to other perceptual dimensions. Why not? It might be argued that color vision is different from 3D vision because it is based (in humans) on the trichromatic mechanisms in the eye that respond differentially to different parts of the electromagnetic spectrum. But having information about the wavelengths of light reflected from a particular surface does not tell us anything about the color (the reflectance characteristics) of that surface because the reflected light is a joint product of the reflectance properties of the surface and the characteristics of the illumination. However, by using the spectral characteristics of the light reflected off a range of surrounding surfaces it is possible to *recover*^4^ the reflectance characteristics of individual surfaces and this has been the basis of several models of color perception including that of Edwin Land. Clearly, such models would fail if we lived in a world of spotlight illumination in which different surfaces are illuminated by different light sources. As a result, it is often claimed that we need to make an *assumption*^4^ about the homogeneity of illumination in order to “*recover*” the reflectance characteristics of surfaces in the scene. But once again, it seems more likely that the mechanisms of our color visual systems have evolved over the millennia to exploit the consequences of the illumination characteristics of our particular world. There is no need to invoke “cognitive” or “higher-level” processes, and this becomes particularly obvious when we think about the visual systems of much simpler animals.

My questioning of words like “cue” and “clue” is merely one aspect of a wider issue—that of the theories we choose to describe the nature of our perceptual system. Traditional theories of perception have assumed that the sensory information is *insufficient* to account for the richness of our perceptions and therefore there is a need to invoke “higher-level” or “cognitive” processes to supplement the inadequate sensory information. Helmholtz (1910) talked about perception being a result of “*unconscious inference,*” Richard Gregory about “*perceptual hypotheses*” and Rock (1984) about “*intelligent, thought-like processes.*”^5^ Clearly, humans are capable of making *inferences* as well as postulating *hypotheses* and being able to *think,* but do such processes affect what we perceive? Do we imagine that our perceptual processes actually make *assumptions* or derive *inferences,* or are we are using these words in a metaphorical sense, that is, *“as if*” there were such processes? Pagel (2019) writes: “*Homuncular language has the air of explanation but it is ultimately explanatorily empty*.”

We also need to ask the question of whether it is possible to distinguish between an evolved perceptual system that has benefitted from a lifetime of perceptual experience and a perceptual system that makes assumptions, derives inferences, and creates hypotheses? One possible distinction is that the use of words like *inference* and *hypotheses* suggests there is an element of choice in what we perceive. For example, Gregory (1966) wrote: “*The visual system entertains alternative hypotheses, and never settles for one solution*” (p. 12) when describing what happens when we view an ambiguous figure like a Necker cube. But the empirical evidence suggests that those alternations occur spontaneously rather than being the result of “higher-level” cognitive processes. Moreover, wouldn't any perceptual system, biological or artificial, suffer from a failure to come up with a unique solution if the input—a wire-frame model of a cube—is ambiguous in terms of the information it provides about its 3D structure? “*The perception is equivocal because what comes to the eye is equivocal*” (Gibson, 1968, p. 247).

The *cognitivisation of perception* and the use of what Pagel (2019) describes as *“homuncular language*” is also relevant when one considers the perceptual systems of animals other than humans. Does it seem likely that fish are capable of making *assumptions* or *inferences*? And if your answer is “no,” is it because we think that the human perceptual system is very different from the perceptual systems of other species? Clearly, humans are different in the sense that we can choose to override what the perceptual information is telling us. For example, a bar of chocolate might appear to be highly desirable when we are hungry but we are capable of ignoring the feeling of hunger and instead choose not to eat it because of concerns about sugar content. But that is about what we choose to do in our *behavior*—rather than a change in what we perceive. Maybe it is this ability to break the normal perception-action loop that is one of the things that distinguishes us from other animals?

## Notes

1. My thanks to Nick Wade for drawing my attention to Helmholtz's use of the word “Zeichen.”

2. “ … die einen gehören der Erfahrung über die besondere Natur der gesehenen Objecte an und geben also nur Vorstellungen des Abstandes,” “… die andern gehören der Empfindung an und geben eine wirkliche Wahrnehmung des Abstandes” p. 623 of the original German edition of *Handbuch der Physiologischen Optik*. Hamburg, Germany: Voss.

3. The word “information” is used in the Gibsonian sense which “*refers to the specification of the observer's environment, not to the specification of the observer's receptors or sense organs.*”

4. Does anything need to be “known,” “recovered,” “assumed,” or “interpreted”? These are examples of words that are probably being used in an “*as if*” sense (see Pagel, 2019 for a fuller discussion).

5. Nine hundred years ago, the Arabic scholar Ibn al-Haytham talked about *“Perception as Inference*” in his 3rd *Book of Optics* (Kitäb al-manäzir (∼1060))*.* (Translated by A. I Sabra, p. 227).
